# Supplementary material for: Dog brains are sensitive to infant- and dog-directed prosody
Source: Commun Biol. 2023 Aug 18;6:859. doi: 10.1038/s42003-023-05217-y (PMC10439206; doi:10.1038/s42003-023-05217-y)
Supplement: Supplementary file 2 — Description of Additional Supplementary Files [file 42003_2023_5217_MOESM2_ESM.pdf]

Description of Additional Supplementary Files

File name: Supplementary Data 1
Description: The source data behind Figure 2- 4.
