# Supplementary material for: Dog brains are sensitive to infant- and dog-directed prosody
Source: Commun Biol. 2023 Aug 18;6:859. doi: 10.1038/s42003-023-05217-y (PMC10439206; doi:10.1038/s42003-023-05217-y)
Supplement: Supplementary file 4 — Reporting Summary [file 42003_2023_5217_MOESM4_ESM.pdf]

nature portfolio

Corresponding author(s): Anna Gergely

Last updated by author(s): Jul 20, 2023

Reporting Summary

Nature Portfolio wishes to improve the reproducibility of the work that we publish. This form provides structure for consistency and transparency in reporting. For further information on Nature Portfolio policies, see our Editorial Policies and the Editorial Policy Checklist.

Statistics

For all statistical analyses, confirm that the following items are present in the figure legend, table legend, main text, or Methods section.

n/a	Confirmed	
☐	☒	The exact sample size (*n*) for each experimental group/condition, given as a discrete number and unit of measurement
☐	☒	A statement on whether measurements were taken from distinct samples or whether the same sample was measured repeatedly
☐	☒	The statistical test(s) used AND whether they are one- or two-sided<br>*Only common tests should be described solely by name; describe more complex techniques in the Methods section.*
☒	☐	A description of all covariates tested
☐	☒	A description of any assumptions or corrections, such as tests of normality and adjustment for multiple comparisons
☐	☒	A full description of the statistical parameters including central tendency (e.g. means) or other basic estimates (e.g. regression coefficient) AND variation (e.g. standard deviation) or associated estimates of uncertainty (e.g. confidence intervals)
☐	☒	For null hypothesis testing, the test statistic (e.g. *F*, *t*, *r*) with confidence intervals, effect sizes, degrees of freedom and *P* value noted<br>*Give P values as exact values whenever suitable.*
☒	☐	For Bayesian analysis, information on the choice of priors and Markov chain Monte Carlo settings
☒	☐	For hierarchical and complex designs, identification of the appropriate level for tests and full reporting of outcomes
☒	☐	Estimates of effect sizes (e.g. Cohen's *d*, Pearson's *r*), indicating how they were calculated

Our web collection on statistics for biologists contains articles on many of the points above.

Software and code

Policy information about availability of computer code

Data collection	Matlab (version 9.1) Psychophysics Toolbox 3
Data analysis	MATLAB R2016b, PM12, PRAAT v.6.1.12, SPSS IBM 22

For manuscripts utilizing custom algorithms or software that are central to the research but not yet described in published literature, software must be made available to editors and reviewers. We strongly encourage code deposition in a community repository (e.g. GitHub). See the Nature Portfolio guidelines for submitting code & software for further information.

Data

Policy information about availability of data

All manuscripts must include a data availability statement. This statement should provide the following information, where applicable:

- Accession codes, unique identifiers, or web links for publicly available datasets
- A description of any restrictions on data availability
- For clinical datasets or third party data, please ensure that the statement adheres to our policy

The authors declare that raw data, including fMRI preprocessing files and analysis of the auditory stimuli are provided in the article (Mendeley Data, doi:https://doi.org/10.17632/76x6zp4j46.1)

Human research participants

Policy information about studies involving human research participants and Sex and Gender in Research.

Reporting on sex and gender	*Use the terms sex (biological attribute) and gender (shaped by social and cultural circumstances) carefully in order to avoid confusing both terms. Indicate if findings apply to only one sex or gender; describe whether sex and gender were considered in study design whether sex and/or gender was determined based on self-reporting or assigned and methods used. Provide in the source data disaggregated sex and gender data where this information has been collected, and consent has been obtained for sharing of individual-level data; provide overall numbers in this Reporting Summary. Please state if this information has not been collected. Report sex- and gender-based analyses where performed, justify reasons for lack of sex- and gender-based analysis.*
Population characteristics	*Describe the covariate-relevant population characteristics of the human research participants (e.g. age, genotypic information, past and current diagnosis and treatment categories). If you filled out the behavioural & social sciences study design questions and have nothing to add here, write "See above."*
Recruitment	*Describe how participants were recruited. Outline any potential self-selection bias or other biases that may be present and how these are likely to impact results.*
Ethics oversight	*Identify the organization(s) that approved the study protocol.*

Note that full information on the approval of the study protocol must also be provided in the manuscript.

Field-specific reporting

Please select the one below that is the best fit for your research. If you are not sure, read the appropriate sections before making your selection.

☒ Life sciences ☐ Behavioural & social sciences ☐ Ecological, evolutionary & environmental sciences

For a reference copy of the document with all sections, see nature.com/documents/nr-reporting-summary-flat.pdf

Life sciences study design

All studies must disclose on these points even when the disclosure is negative.

Sample size	In the present study we applied non-invasive fMRI on family dogs. All of them completed a special training (developed by one of the authors of this paper), that was based on the model-rival technique and included elements of conditioning and social learning, applying positive reinforcement, in order to teach dogs to stay still in the scanner. Sample size of the present study (N=19) can be considered as sufficient and/or large in comparison to previously published canine non-invasive fMRI studies with N=2-13 sample sizes (e.g. Berns 2012, Andics et al 2014, 2016).
Data exclusions	Inclusion criteria for the fMRI scan for each dog and each runs were 3 mm and 3° for each direction. If head movements exceeded this threshold the affected and all following volumes of the scan were excluded (55 from 57 functional runs were not affected by these exclusion criteria; volume 26-32 of a run of Dog08 and volume 27-32 of a run of Dog11 were excluded.
Replication	We did not replicate our results as private owners of these specially trained family dogs had limited time, therefore we have limited access to the sample dogs.
Randomization	In all three fMRI runs, stimuli conditions were counterbalanced to speakers' gender (i.e. in one run 4 stimuli were used from male and 4 from female speakers in each condition). One speaker's voice was used only once per run. The run order was also counterbalanced across dogs.
Blinding	Experimenters were blind to the the run number, stimulus order etc. that the dog was exposed to. Only MRI assistant colleagues were aware of the scanning protocol, who were not involved to the data analysis.

Reporting for specific materials, systems and methods

We require information from authors about some types of materials, experimental systems and methods used in many studies. Here, indicate whether each material, system or method listed is relevant to your study. If you are not sure if a list item applies to your research, read the appropriate section before selecting a response.

Materials & experimental systems

n/a	Involved in the study
☒	☐ Antibodies
☒	☐ Eukaryotic cell lines
☒	☐ Palaeontology and archaeology
☐	☒ Animals and other organisms
☒	☐ Clinical data
☒	☐ Dual use research of concern

Methods

n/a	Involved in the study
☒	☐ ChIP-seq
☒	☐ Flow cytometry
☐	☒ MRI-based neuroimaging

Animals and other research organisms

Policy information about studies involving animals; ARRIVE guidelines recommended for reporting animal research, and Sex and Gender in Research

Laboratory animals
This study did not involve any laboratory animals. Only specifically-trained adult family dogs, owned by private owners, were measured in the present experiment.

Wild animals
Provide details on animals observed in or captured in the field; report species and age where possible. Describe how animals were caught and transported and what happened to captive animals after the study (if killed, explain why and describe method; if released, say where and when) OR state that the study did not involve wild animals.

Reporting on sex
Indicate if findings apply to only one sex; describe whether sex was considered in study design, methods used for assigning sex. Provide data disaggregated for sex where this information has been collected in the source data as appropriate; provide overall numbers in this Reporting Summary. Please state if this information has not been collected. Report sex-based analyses where performed, justify reasons for lack of sex-based analysis.

Field-collected samples
For laboratory work with field-collected samples, describe all relevant parameters such as housing, maintenance, temperature, photoperiod and end-of-experiment protocol OR state that the study did not involve samples collected from the field.

Ethics oversight
Ethical approval for this study was obtained from the National Scientific Ethical Committee on Animal Experimentation (PEI/001/1057-6/2015). Owners volunteered with their dogs to participate in the study, did not get any monetary compensation and gave a written informed consent.

Note that full information on the approval of the study protocol must also be provided in the manuscript.

Magnetic resonance imaging

Experimental design

Design type
auditory fMRI, block design

Design specifications
N=19 trained dogs; 3, approximately 7-minute-long runs during 1-3 sessions per subject, 8-second-long blocks between 2-second-long volume acquisitions; 32 blocks per run

Behavioral performance measures
There were no behavioral measures.

Acquisition

Imaging type(s)
functional

Field strength
3T

Sequence & imaging parameters
single-shot gradient-echo planar imaging (EPI) sequence, volumes : 31, 2.0 mm thick coronal slices in a R >> L sequence; TE: 30.0 ms; TR: 10000 ms; flip angle: 90°; acquisition matrix: 64×64

Area of acquisition
whole brain

Diffusion MRI ☐ Used ☒ Not used

Preprocessing

Preprocessing software
Image preprocessing and statistical analysis were performed using MATLAB R2016b and SPM12. First, we realigned functional images of the 3 runs in case of each dog. Second, to correct for the different orientation of human and dog heads in the scanner, we manually reoriented each mean-functional and realigned-functional files. Third, we transformed mean-functional images for each dog to the template space via Amira 3D software platform. Fourth, we made a second normalization in spm12, in which we added the normalized-mean-functional files (the ones transformed in Amira 3D) as a template, the original non-normalized-mean-functional files as a source image and we applied the transformation matrix

	between the two previous images for all realigned-functional files, that resulted all images to be in a shared space. Finally, we convolved with an isotropic 3-D Gaussian kernel (FWHM = 4 mm) for spatial filtering of all normalized-functional files. We centered all images around the Commissura rostralis.
Normalization	General Linear Model and statistical parametric mapping were used for data analyses. Our model was built with condition regressors for the 7 conditions (FADS, FDDS, FIDS, MADS, MDDS, MIDS, SIL modeled as a 6.5 long blocks) and for the 3 runs. Single-subject fixed effect analyses were followed by whole-volume random effects analyses on the group level. We searched for effects of addressee (ADS, DDS and IDS), speaker's gender (F, M) and addressee × gender interaction within the whole brain.
Normalization template	As a brain template for analyses we used the anatomical image of a golden retriever's brain (Czeibert et al., 2019).
Noise and artifact removal	To the normalization, we added potential movement artifacts as regressors, resulting from the realignment step. To filter out the potential muscle-movement artifacts, hailed from the tight muscle-tissue around the dogs' head, we used a whole-brain inclusive mask in the individual-level analyses.
Volume censoring	Overall motion/rotation threshold was 3 mm and 3° for each direction.

Statistical modeling & inference

Model type and settings	Single-subject fixed effect analyses were followed by whole-volume random effects analyses on the group level.
Effect(s) tested	We searched for effects of addressee (ADS, DDS and IDS), speaker's gender (F, M) and addressee × gender interaction within the whole brain.

Specify type of analysis: ☐ Whole brain ☐ ROI-based ☒ Both

Anatomical location(s): L c/rSG: left caudo-rostral transition of the Sylvian gyrus; L cSG/TP: a region involving the most ventral part of the caudal Sylvian gyrus and the temporal pole

Statistic type for inference (See Eklund et al. 2016)	cluster-wise
Correction	FWE

Models & analysis

n/a	Involved in the study
☒	☐ Functional and/or effective connectivity
☒	☐ Graph analysis
☒	☐ Multivariate modeling or predictive analysis
